# Sampling technique biases in the analysis of fruit fly volatiles: a case study of Queensland fruit fly

**DOI:** 10.1038/s41598-020-76622-0

**Published:** 2020-11-13

**Authors:** Saeedeh Noushini, Soo Jean Park, Ian Jamie, Joanne Jamie, Phillip Taylor

**Affiliations:** 1 Department of Molecular Sciences, Macquarie University, Sydney, NSW 2109 Australia; 2 Applied BioSciences, Macquarie University, Sydney, NSW 2109 Australia; 3 Australian Research Council Industrial Transformation Training Centre for Fruit Fly Biosecurity Innovation, Macquarie University, Sydney, NSW 2109 Australia

**Keywords:** Chemical biology, Chemistry

## Abstract

Diverse methods have been used to sample insect semiochemicals. Sampling methods can differ in efficiency and affinity and this can introduce significant biases when interpreting biological patterns. We compare common methods used to sample tephritid fruit fly rectal gland volatiles (‘pheromones’), focusing on Queensland fruit fly, *Bactrocera tryoni*. Solvents of different polarity, *n*-hexane, dichloromethane and ethanol, were compared using intact and crushed glands. Polydimethylsiloxane, polydimethylsiloxane/divinylbenzene and polyacrylate were compared as adsorbents for solid phase microextraction. Tenax-GR and Porapak Q were compared as adsorbents for dynamic headspace sampling. Along with compounds previously reported for *B. tryoni*, we detected five previously unreported compounds in males, and three in females. Dichloromethane extracted more amides while there was no significant difference between the three solvents in extraction of spiroacetals except for (*E*,*E*)-2,8-dimethyl-1,7-dioxaspiro[5.5]undecane for which *n*-hexane extracted higher amount than both dichloromethane and ethanol. Ethanol failed to contain many of the more volatile compounds. Crushed rectal gland samples provided higher concentrations of extracted compounds than intact rectal gland samples, but no compounds were missed in intact samples. Of solid phase microextraction fibers, polyacrylate had low affinity for spiroacetals, ethyl isobutyrate and ethyl-2-methylbutanoate. Polydimethylsiloxane was more efficient for spiroacetals while type of fiber did not affect the amounts of amides and esters. In dynamic headspace sampling, Porapak was more efficient for ethyl isobutyrate and spiroacetals, while Tenax was more efficient for other esters and amides, and sampling time was a critical factor. Biases that can be introduced by sampling methods are important considerations when collecting and interpreting insect semiochemical profiles.

## Introduction

Semiochemicals, including pheromones, are of central importance in the biology of many insects, including tephritid fruit flies. Because semiochemicals are commonly produced and released at low concentrations, efficient sampling methods are needed for collection and subsequent identification and quantification^1–5^. Tephritid fruit flies typically store pheromones in rectal glands and release them into the air during sexual activity^4,6–13^. Diverse sampling methods have been used to sample fruit fly pheromones and, in addition to genuine biological differences, some variation in pheromones reported for different fruit flies may actually arise from differences in the chemical collection efficiencies of the sampling methods used. The most common method entails immersion of rectal glands in organic solvents. Common solvents that have been used for fruit fly rectal gland extraction vary in polarity and include *n*-pentane, *n*-hexane, acetone, dichloromethane and ethanol^12,14–17^. In some studies the glands have been intact while in others the glands have been crushed^18–20^.

Rather than focusing on compounds stored in the rectal glands, some studies have instead focused on collecting the emitted volatiles. While this approach does not identify the glandular source of the emissions, it has the advantage of being a whole-animal method, thus detecting volatiles that might be produced and emitted by glands other than the rectal glands, and hence more fully represents the array of compounds, and blends, that might be encountered by receivers. In most studies, emitted volatiles are trapped onto an adsorbent material using either dynamic or static sampling techniques^21^. Dynamic headspace sampling techniques involve passing an airflow to purge and trap volatiles onto an adsorbent material such as Porapak (ethylvinylbenzene-divinylbenzene copolymer), activated charcoal, or Tenax (porous polymer based on 2,6-diphenyl-*p*-phenylene oxide). Tenax^15,22,23^ and Porapak Q^24,25^ have been widely used in sampling of fruit fly pheromones. Static headspace sampling techniques involve use of adsorbent materials without airflow. The most commonly used static sampling method utilizes SPME (solid phase microextraction) adsorbent fibers, such as polydimethylsiloxane (PDMS), carboxen (CAR), divinylbenzene (DVB), polyacrylate (PA), or a mixed-phase coating, which vary in efficiency depending on the polarity of targeted compounds. PDMS fibers are widely used for collection of non-polar compounds^6,26–28^. Polyacrylate (PA) has a high affinity to more polar compounds and hence has been used for polar semiochemicals^29,30^. PDMS/divinylbenzene (DVB), a mixed-phase coating that covers a broader spectrum due to their distinct polarity, has also been used for collection of semiochemicals^19,20,29,31–33^. Because adsorbent materials differ in affinity for particular groups of semiochemicals, a poor choice of material can result in substantial under-sampling, or even failure to even detect some compounds.

*Bactrocera tryoni* is the most economically important pest fruit fly in Australia^34,35^, being highly polyphagous and attacking most fruit crops^36^. Volatile profiles of male and female *B. tryoni* have been described previously^19,37–39^. Similar to many other fruit flies, *B. tryoni* stores secreted volatiles in the rectal glands^7,8,40,41^. *Bactrocera tryoni* mating is limited to a period of about 30 min at dusk^37,41,42^. During calling and courtship, males release the sweet-smelling volatile blend containing six aliphatic amides that have been generally interpreted as sex pheromones^7,37,41–45^. Although the functions of the individual components of the male *B. tryoni* sex pheromone blend have not been studied, virgin mature females are attracted to volatiles from crushed male glands or calling males^43,44^. The secretions reported for *B. tryoni* females have differed somewhat between studies, and this may reflect differences in sampling methods^19,38^. Booth et al.^19^ reported a diverse suite of spiroacetals as predominant compounds from *n*-pentane extracts of the whole crushed abdomen, while El-Sayed et al.^38^ found saturated/unsaturated esters as predominant compounds from *n*-hexane extracts of intact rectal glands. The contrast between these studies may indicate that while spiroacetals are not present in the rectal glands they may be produced in other glands elsewhere in the abdomen^38^.

The present study considers the effects of sampling methods on the detection and quantification of fruit fly volatiles, using *B. tryoni* as a model species. The main purpose of this study was to highlight advantages and disadvantages of the different methods adopted for rectal gland extractions and headspace collection. We investigated the effect of (1) solvent polarity, (2) crushing of sampled glands in solvent, (3) adsorbent types in both dynamic and static sampling techniques, and (4) volume of air sampled in dynamic sampling techniques. In addition, we identified six previously unreported compounds in male *B. tryoni* rectal gland contents/emissions and three in females. These compounds resolve a long-standing discord between the perceptible odor and known blend composition in *B. tryoni*.

## Materials and methods

### Insects

All experiments were conducted using *B. tryoni* from a laboratory culture at Macquarie University, Sydney, Australia (originating from central coastal New South Wales, G27). Adults were provided sugar and yeast hydrolysate (MP Biomedicals LLC) as food, and tap water through a soaked sponge. Virgin male and female flies were segregated within 4 days after eclosion, transferred to 12.5 L clear plastic cages (180 flies per cage) and maintained in controlled environment rooms (25 ± 0.5 °C, 65 ± 5% relative humidity (RH) and 11.5: 0.5: 11.5: 0.5 light/dusk/dark/dawn photoperiod) until they were used in experiments. No calling or mating was observed prior to separating the sexes. Flies used for all experiments were 13—18 days old (sexually mature) virgins (see Perez-Staples et al.^46^).

### Chemicals

*n*-Hexane, dichloromethane, ethanol and the following chemicals were purchased from Sigma-Aldrich (St Louis, MO, US), Alfa-Aesar (Ward Hill, MA, US), Chem-Supply (Bedford St, Gillman, SA) and Nu-Chek-Prep and INC (Elysian, MN, US), with the purities noted in parentheses, and were used without further purification: hexadecane (99%), ethyl propanoate (99%), ethyl isobutyrate (≥ 98%), ethyl 2-methylbutanoate (99%), propyl isobutyrate (≥ 97%), ethyl 2-methylpentanoate (≥ 98%), diethyl succinate (99%), methyl laurate (≥ 98%), ethyl laurate (≥ 98%), methyl myristate (≥ 98%), ethyl myristate (99%), ethyl myristoleate (97%), methyl palmitoleate (≥ 99%), ethyl palmitate (≥ 99%) and ethyl oleate (98%). Propyl laurate, ethyl palmitoleate, ethyl elaidate, *N*-(2-methylbutyl)acetamide, *N*-(3-methylbutyl)acetamide, *N*-(2-methylbutyl)propanamide, *N*-(3-methylbutyl)propanamide, *N*-(2-methylbutyl)isobutyrate and *N*-(3-methylbutyl)isobutyrate and (*E*,*E*)-2,8-dimethyl-1,7-dioxaspiro[5.5]undecane were synthesized (see Supplementary Information for synthesis details). 2,7-Dimethyl-1,6-dioxaspiro[4.5]decane^47^, (*E*,*E*)-2-ethyl-8-methyl-1,7-dioxaspiro[5.5]undecane^18^, (*E*,*Z*)-2-ethyl-7-methyl-1,6-dioxaspiro[4.5]decane^32^, (*E*,*E*)-2-ethyl-2,8-dimethyl-1,7-dioxaspiro[5.5]undecane^19^ and (*E*,*E*)-2-propyl-8-methyl-1,7-dioxaspiro[5.5]undecane^32^, were tentatively identified based on literature mass spectral fragmentation patterns (Table 1).

**Table 1:** Compounds produced by adults of *Bactrocera tryoni*. * Compounds identified in males, # Compounds identified in females, No = Number, I = Identification, AS = authentic sample, L = literature, KI = Kovats index.

No	Name	Characteristic EI ions *m/z* (%)	I	KI
1	Ethyl propanoate*	102 (M^+^, 10), 74 (14.5), 57 (100)	AS	672
2	Ethyl isobutyrate*	116 (M^+^, 24.8), 88 (43.9), 71 (100)	AS	727
3	Ethyl 2-methylbutanoate*	130 (M^+^, 1.23), 115 (8.0), 102 (60.8), 85 (37.9), 74 (25.6), 57 (100)	AS	844
4	Propyl isobutyrate*	130 (M^+^, 0.5), 102 (8.2), 101 (5.9), 89 (83.7), 71 (100)	AS	850
5	Ethyl 2-methylpentanoate*	144 (M^+^, 2.9), 115 (M – C_2_H_5_, 9.9), 102 (67.5), 99 (M – OC_2_H_5_, 18.1), 74 (41.3), 55 (23.5), 45 (100)	AS	933
6	*N*-(2-Methylbutyl)acetamide*^#^	129 (M^+^, 6.0), 114 (M – CH_3_, 12.2), 100 (M – C_2_H_5_, 52.3), 73 (β-cleavage/H rearrangement, 57.4), 72 (M – C_4_H_9_,100), 60 (CH_3_C(OH)NH^+^, 60.3), 58 (27.9), 55 (16.1)	AS	1123
7	*N*-(3-Methylbutyl)acetamide*^#^	129 (M^+^, 5.3), 114 (M – CH_3_, 16.0), 86 (M – C_3_H_7_, 29.2), 73 (β-cleavage/H rearrangement, 100), 72 (M – C_4_H_9_, 74.7), 60 (CH_3_C(OH)NH^+^, 30.1), 55 (17.3)	AS	1129
8	Diethyl succinate*	174 (M^+^, 0.4), 129 (M – OC_2_H_5_, 57.6), 128 (20.1), 101 (M – COOC_2_H_5_, 100), 73 (21.1), 74 (11.2), 55(15.6)	AS	1172
9	*N*-(2-Methylbutyl)propanamide*^#^	143 (M^+^, 8.0), 114 (M – C_2_H_5_, 15.4), 87 (β-cleavage/H rearrangement, 50.6), 86 (M – C_4_H_9_, 100), 74 (CH_3_CH_2_C(OH)NH^+^, 83.1), 58 (39.7), 57(95.0)	AS	1198
10	*N*-(3-Methylbutyl)propanamide*^#^	143 (M^+^, 4.8), 128 (M – CH_3_, 10.4), 114 (M – C_2_H_5_, 16.3), 100 (15.2), 87 (β-cleavage/H rearrangement, 100), 86 (M – C_4_H_9_, 62.9), 74 (CH_3_CH_2_C(OH)NH^+^, 28.1), 57 (66.6)	AS	1204
11	*N*-(2-Methylbutyl)isobutyrate*	157 (M^+^, 9.0), 128 (M – C_2_H_5_, 15.3), 114 (M – C_3_H_7_, 10.2), 101 (β-cleavage/H rearrangement, 15.2), 100 (M – C_4_H_9_, 16.2) 88 (CH_3_CHCH_3_C(OH)NH^+^, 77.2), 71 (100)	AS	1226
12	*N*-(3-Methylbutyl)isobutyrate*^#^	157 (M^+^, 9.5), 142 (M – CH_3_, 17.2), 114 (M – C_3_H_7_, 23.6), 101 (β-cleavage/H rearrangement, 75.6), 100 (M – C_4_H_9_, 20.1), 88 (CH_3_CHCH_3_C(OH)NH^+^, 27.0), 71 (100)	AS	1230
13	2,7-Dimethyl-1,6-dioxaspiro[4.5]decane^#^	170 (M^+^, 0.3), 155 (M – CH_3_, 0.8), 126 (17.4), 115 (12.8), 111 (4.2), 101 (CH_3_(C_4_H_5_O) = OH^+^, 100), 98 (CH_3_(C_4_H_5_O) = CH_2_, 88.7), 83 (43.8), 69 (29.4), 55 (52.7)	L^47^	1076
14	(*E*,*E*)-2,8-Dimethyl-1,7-dioxaspiro[5.5]undecane^#^	184 (M^+^, 9.4), 169 (M – CH_3_, 1.8), 140 (12.7), 125 (8.1), 115 (CH_3_(C_5_H_7_O) = OH^+^, 86.5), 112 (CH_3_(C_5_H_7_O) = CH_2_, 100), 97 (61.2), 69 (46.6), 55 (43.1)	AS	1148
15	(*E*,*E*)-2-Ethyl-8-methyl-1,7-dioxaspiro[5.5]undecane^#^	198 (M^+^, 10.9), 169 (M—C_2_H_5_, 11.6), 140 (15.2), 129 (CH_3_CH_2_(C_5_H_7_O) = OH^+^, 47.7), 126 (CH_3_CH_2_(C_5_H_7_O) = CH_2_, 43.6), 115 (CH_3_(C_5_H_7_O) = OH^+^, 100), 112 (CH_3_(C_5_H_7_O) = CH_2_, 90.7), 97 (59.2), 83 (51.6), 69 (71.3), 55 (70.8)	L^18^	1239
16	(*E*,*Z*)-2-Ethyl-7-methyl-1,6-dioxaspiro[4.5]decane^#^	184 (M^+^, 1.1), 168 (0.8), 155 (M – C_2_H_5_, 13.2), 140 (1.5), 115 (CH_3_(C_5_H_7_O) = OH^+^ and CH_3_CH_2_(C_4_H_5_O) = OH^+^, 100), 112 (CH_3_CH_2_(C_5_H_7_O) = CH_2_ and CH_3_CH_2_(C_4_H_5_O) = CH_2_, 41.8), 97 (91.7), 85 (9.1), 83 (8.3), 69 (85.8), 55 (54.9)	L^32^	1275
17	(*E*,*E*)-2-Ethyl-2,8-dimethyl-1,7-dioxaspiro[5.5]undecane^#^	212 (M^+^, 2.6), 183 (M – C_2_H_5_, 12.8), 143 (CH_3_(C_5_H_6_O) = OHCH_2_CH_3_^+^, 57.2), 140 (CH_3_(C_5_H_6_O) = CH_2_CH_2_CH_3_, 11.2), 125 (CH_2_CH(C_5_H_7_O)CH_3_,100), 115 (CH_3_(C_5_H_7_O) = OH^+^, 46.3),112 (CH_3_(C_5_H_7_O) = CH_2_, 70.7), 97 (57.6), 82 (55.6), 83 (60.6), 55 (75.1)	L^19^	1318
18	(*E*,*E*)-2-Propyl-8-methyl-1,7-dioxaspiro[5.5]undecane^#^	212 (M^+^, 7.3), 169 (M – C_3_H_7_, 13.2), 143 (CH_3_CH_2_CH_2_(C_5_H_6_O) = OH^+^, 31.5), 140 (CH_3_CH_2_CH_2_(C_5_H_6_O) = CH_2_CH_2_CH_3_, 36.5), 125 (44.5), 115 (CH_3_(C_5_H_7_O) = OH^+^, 100),112 (CH_3_(C_5_H_7_O) = CH_2_, 73.4), 97 (72.1), 82 (26.2), 83 (34.6), 69 (46.8), 55 (69.2)	L^32^	1324
19	Methyl laurate^#^	214 (M^+^, 3.68), 183 (M – OCH_3_, 8.0), 171 (14.6), 143 (18.2), 129 (9.5), 87 (60.1), 74 (McLafferty rearrangement product, 100), 59 (COOCH_3_, 8.5), 55 (22.8)	AS	1524
20	Ethyl laurate^#^	228 (M^+^, 4.3), 199 (M – C_2_H_5_, 4.7), 183 (M – OC_2_H_5_, 5.6), 157 (7.6), 101 (35.9), 88 (McLafferty rearrangement product, 100), 73 (COOC_2_H_5_, 20.7), 70 (21.8), 61 (14.9), 60 (13.7), 55 (27.0)	AS	1595
21	Propyl laurate^#^	242 (M^+^, 1.6), 201 (40.4), 199 (M – C_3_H_7_, 1.0), 183 (M – OC_3_H_7_, 36.5), 157 (9.2), 129 (15.2), 115 (26.7), 102 (McLafferty rearrangement product, 29.7), 87 (COOC_3_H_7_, 11.2), 61 (100), 59 (4.1), 57 (30), 55 (26.0)	AS	1691
22	Methy myristate^#^	242 (M^+^, 6.6), 211 (M – OCH_3_, 6.3), 199 (16.2), 143 (25.6), 125 (1.1), 111 (2.6), 101 (8.8), 97 (6.3), 87 (64.4), 74 (McLafferty rearrangement product, 100), 59 (COOCH_3_, 7.8), 55 (23.4)	AS	1727
23	Ethyl myristate^#^	256 (M^+^, 7.1), 213 (13.8), 211 (M – OC_2_H_5_, 8.1), 157 (22.0), 101 (53.8), 88 (McLafferty rearrangement product, 100), 73 (COOC_2_H_5_, 17.8), 70 (22.1), 55 (20.1)	AS	1795
24	Ethyl myristoleate^#^	254 (M^+^, 4.1), 209 (M – OC_2_H_5_, 13.9), 208 (M – C_2_H_5_OH, 15.0), 155 (9.3), 166 (28.9), 124 (23.7), 88 (McLafferty rearrangement product, 46.4), 73 (COOC_2_H_5_, 16.6), 69 (25.2), 55 (100)	AS	1785
25	Methyl palmitoleate^#^	268 (M^+^, 5.1), 237 (M – OCH_3_, 14.2), 236 (M – CH_3_OH, 18.6), 194 (18.0), 152 (24.1), 97 (51.6), 96 (51.4), 74 (McLafferty rearrangement product, 52.3), 69 (63.6), 59 (COOCH_3_, 17.1), 55 (100)	AS	1909
26	Ethyl palmitoleate^#^	282 (M^+^, 6.7), 237 (M – OC_2_H_5_, 14.0), 236 (M – C_2_H_5_OH, 21.3), 194 (23.2), 152 (28.6), 88 (McLafferty rearrangement product, 57.3), 73 (COOC_2_H_5_, 16.8), 69 (68.7), 55 (100)	AS	1977
27	Ethyl palmitate^#^	284 (M^+^, 11.3), 255 (M – C_2_H_5_, 4.1), 241 (13.2), 239 (M – OC_2_H_5_, 7.5), 157 (21.3), 115 (8.4), 101 (57.5), 88 (McLafferty rearrangement product, 100), 73 (COOC_2_H_5_, 16.2), 55 (21.1)	AS	1995
28	Ethyl oleate^#^	310 (M^+^, 1.2), 265 (M – OC_2_H_5_, 3.8), 264 (M – C_2_H_5_OH, 8.170), 222 (5.4), 180 (5.0), 125 (13.6), 123 (13.6), 111 (18.5), 97 (39.8), 88 (McLafferty rearrangement product, 35.4), 83 (50.1), 73 (COOC_2_H_5_, 15.2), 69 (77.1), 55 (100)	AS	2144
29	Ethyl elaidate^#^	310 (M^+^, 0.5), 265 (M – OC_2_H_5_, 4.6), 264 (M – C_2_H_5_OH, 9.3), 222 (5.8), 180 (5.4), 123 (14.1), 110 (18.7), 97 (38.2), 88 (McLafferty rearrangement product, 33.2), 83 (44.4), 73 (COOC_2_H_5_, 13.6), 69 (70.1), 55 (100)	AS	2172

### GC–MS analysis

Gas Chromatography-Mass Spectrometry (GC–MS) analyses were performed using a Shimadzu GCMS-QP2010 or GCMS-TQ8040 instrument, which was equipped with a capillary column with 5% diphenyl/95% dimethyl polysiloxane as the stationary phase (30 m × 0.25 mm I.D. × 0.25 μm film thickness). Helium (99.999%, BOC, North Ryde, NSW, Australia) at a flow rate of 1.0 mL/min was used as a carrier gas. The oven temperature was held at 50 °C for 4 min or 40 °C for 1 min then programmed at 10 °C/min to 250 °C, with splitless injection mode at 270 °C. The temperatures of interface and ion source were 290 and 200 °C, respectively. Mass detection was performed in EI mode at a voltage of 70 eV. The spectra were obtained over a mass range of 45 to 500 m*/z*. The condition used for determining the Kovats retention index was the same as above, with the oven initial temperature at 40 °C for 1 min.

### Rectal gland samples

*n*-Hexane, dichloromethane (DCM) and ethanol (EtOH) were used for separate rectal gland extractions. For each solvent 10 replicates containing 10 glands were collected for each sex. Flies were first killed by chilling them on dry ice 3 – 5 h before the onset of dusk. Rectal glands were extracted by gently pressing the abdomen and pulling the gland out with fine forceps. Glands were carefully placed in a 1.1 mL tear-drop vial in dry ice. Once 10 glands were collected, the vials were removed from the dry ice and 100 µL of solvent was added. Glands were saturated with solvent at room temperature for 10 min The extracts were then transferred to a new vial and stored at -20 °C until analyzed. Hexadecane was used as an internal standard, with 2 µL of 1.35 mg/mL stock solution being added to each extract. To assess the effects of crushing the glands, an additional 10 samples were assessed using *n*-hexane as a solvent. For these samples, when 100 µL of *n*-hexane was added, the 10 rectal glands were crushed using a capillary glass tube. Other steps were as for the intact gland samples. Gloves (Ni-Tek) were used when collecting and handling samples to minimize risk of contamination.

### Headspace samples

#### Dynamic method

Tenax-GR Mesh 60/80, 50 mg (Scientific Instrument Services, Inc) and Porapak Q 80–100 mesh, 50 mg (Waters, USA) were packed into 6 × 50 mm glass cartridges and held in place with glass wool plugs (Table S1). Tenax and Porapak were separately conditioned under nitrogen (75 mL/min) at 200 °C and 180 °C respectively for three hours before each sample collection. For each collection, 30 males or 30 females were placed into a glass chamber (150 mm long and 40 mm ID) 30 min before dusk to acclimatize. Dusk in the controlled environment room was simulated for 30 min. While the period of active calling is well known from observations of wing fanning behaviour^37,41,42^ it is possible that emissions continue beyond this time. Charcoal-filtrated air (0.5 L/min, air pulling system) was passed over the flies for 10 min and 20 min, starting from the end of dusk phase, 40 min, 60 min and 90 min, starting from beginning of dusk, to cover all likely release times. Volatiles were subsequently eluted from Tenax or Porapak using 1 mL *n*-hexane, and concentrated to 200 µL under a gentle nitrogen stream. Six replicates per sex per sampling period were collected for each sorbent. Samples were stored at -20 °C until analyzed. To distinguish between volatile compounds released by the flies and possible contaminants, an air control sample comprising an empty glass chamber was run and analyzed along with each headspace collection. Hexadecane was then used as an internal standard, with 2 µL of 2.7 mg/mL stock solution being incorporated to each concentrated samples. 1 µL of each sample was injected for GC–MS analysis.

#### Static method

A manual holder (Supelco, Bellefonte, PA, US) was used with three different fibers; 100 µm film thickness PDMS, 65 µm PDMS–DVB and 85 µm PA (Table S1). Fibers were thermally conditioned in the GC injection port for 30 min at 270 °C. Six replicates per sex were carried out for each fiber. For each replicate, 5 males or females were placed in a 40 mL clear glass vial 30 min before dusk to acclimatize. When dusk was finished, the fiber was exposed for 10 min, which was sufficient time for adsorption of volatiles without saturating the fiber. The loaded SPME fiber was then injected into the GC–MS.

Prior to each headspace collection, glass chambers and vials were washed with 5% Extran aqueous solution, rinsed with hot tap water, and heated at 200 ºC for 18 h. Activated charcoal filters were thermally conditioned by heating them at 200 °C for 18 h prior to each headspace collection^48^. To distinguish any possible contaminants, an air control sample comprising an empty glass vial was run and analyzed along with each headspace collection.

## Data analysis

Normalized GC peak areas were used to compare the proportions of each compound. Normalized peak areas were obtained by dividing the peak area of interested compounds by the peak area of the internal standard. The data sets were not normally distributed and were transformed to log (x + 1) for statistical analysis. All graphics were generated using normalized GC peak areas without log-transformation, except for SPME, for which the actual peak areas were used. The effects of sampling method were analyzed by ANOVA, followed by a Tukey post hoc test (α = 0.05) for multiple comparisons using SPSS (IBM Corp. released 2012 IBM SPSS Statistics for Windows, v. 21.0. Armonk, NY, IBM Corp.). When comparing the extraction efficiency of different solvents of rectal gland contents, solvent and compound were fixed factors. When comparing extractions from intact and crushed rectal glands in *n-*hexane, crushing treatment and compound were fixed factors. When comparing SPME fibers for static headspace sampling, fiber and compound were fixed factors. When comparing sorbents and collection time for active headspace sampling, sorbent, compound and time were fixed factors. Due to low concentrations, ethyl propanoate, propyl isobutyrate and ethyl 2-methylpentanoate were excluded from statistical analysis of male rectal glands and headspace while propyl laurate and (*E*,*Z*)-2-ethyl-7-methyl-1,6-dioxaspiro[4.5]decane were excluded from statistical analysis of female rectal glands and headspace. For the analysis of amides, the peak area from male samples were used for statistical analysis as females produced the same amides as males but in lower concentration.

## Results

A full listing of the compounds identified in rectal gland extracts and emissions of *B. tryoni* is provided in Table 1. Twenty two compounds were detected in rectal gland extracts and headspace collections of sexually mature virgin female *B. tryoni*, including five amides (**6**, **7**, **9**, **10** and **12**), six spiroacetals (**13**–**18**) and eleven esters (**19**–**29**). Of these, 19 compounds were detected in the headspace. Ethyl oleate (**28**), ethyl elaidate (**29**) and *N*-(2-methylbutyl)acetamide (**6**) were not detected in the headspace. Of the 22 compounds detected in our study, three minor compounds, *N*-(2-methylbutyl)acetamide (**6**), propyl laurate (**21**) and methyl myristate (**22**) have not been previously reported in *B. tryoni*.

Twelve compounds were detected in rectal gland extracts and headspace collections of male *B. tryoni*, including the seven previously reported compounds, *N*-(2-methylbutyl)acetamide, *N*-(3-methylbutyl)acetamide, *N*-(2-methylbutyl)propanamide, *N*-(3-methylbutyl)propanamide, *N*-(2-methylbutyl)isobutyrate, *N*-(3-methylbutyl)isobutyrate and ethyl isobutyrate and five additional compounds that have not been previously reported (Table 1); ethyl propanoate, ethyl 2-methylbutanoate, propyl isobutyrate, ethyl 2-methylpentanoate and diethyl succinate.

### Rectal gland extractions

#### Effect of solvent

**Figure 1:**
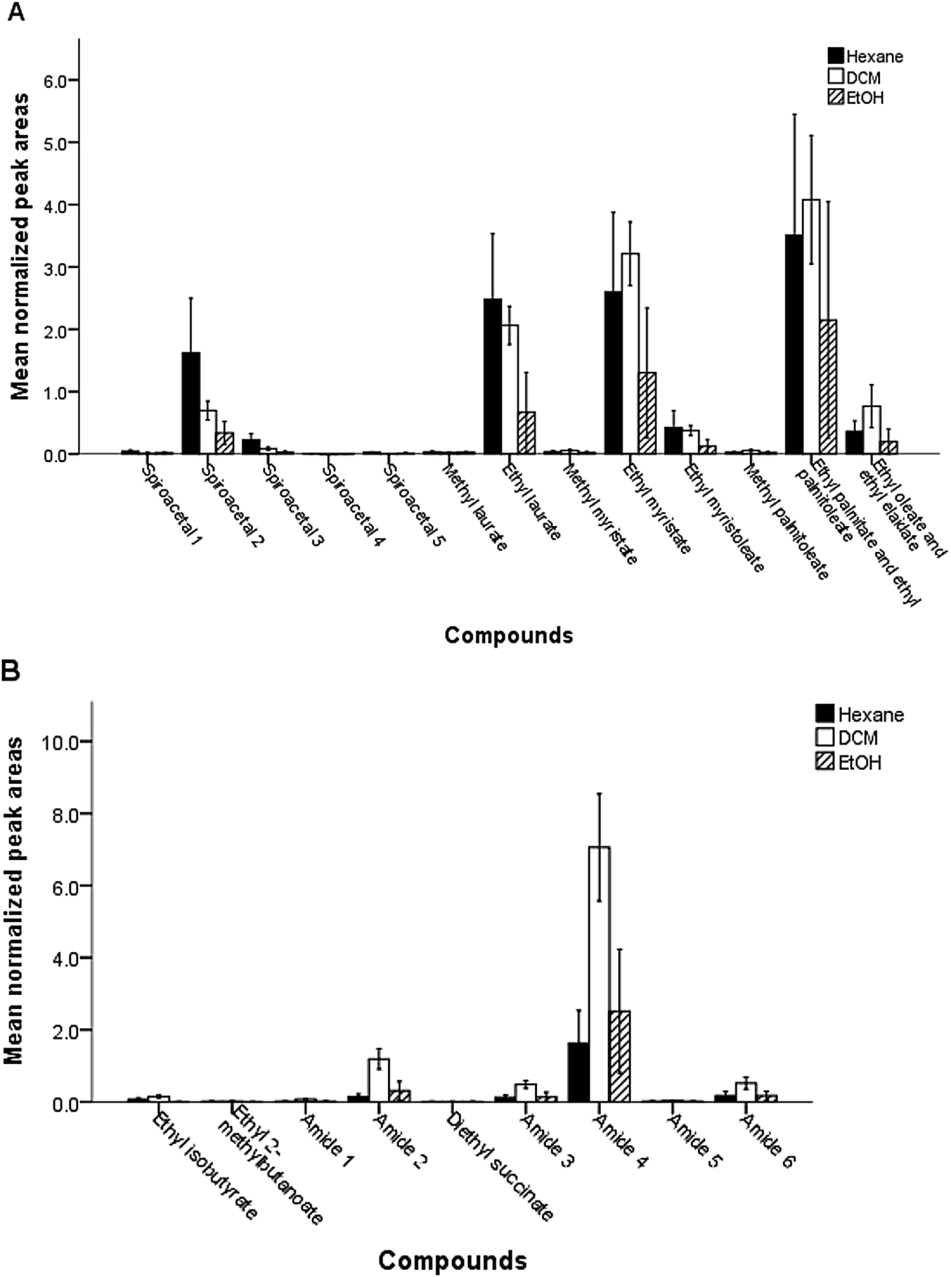
Graphical display of mean normalized peak areas (*n* = 10) obtained for rectal glands extract of female (**A**) and male (**B**) *Bactrocera tryoni* using three different solvents, *n*-hexane, dichloromethane (DCM) and ethanol (EtOH). Error bars represent the confidence interval for the mean at 95% confidence level. Amide 1: *N*-(2-methylbutyl)acetamide, Amide 2: *N*-(3-methylbutyl)acetamide, Amide 3: *N*-(2-methylbutyl)propanamide, Amide 4: *N*-(3-methylbutyl)propanamide, Amide 5: *N*-(2-methylbutyl)isobutyrate, Amide 6: *N*-(3-methylbutyl)isobutyrate, Spiroacetal 1: 2,7-dimethyl-1,6-dioxaspiro[4.5]decane, Spiroacetal 2: (*E*,*E*)-2,8-dimethyl-1,7-dioxaspiro[5.5]undecane, Spiroacetal 3: (*E*,*E*)-2-ethyl-8-methyl-1,7-dioxaspiro[5.5]undecane, Spiroacetal 4: (*E*,*E*)-2-ethyl-2,8-dimethyl-1,7-dioxaspiro[5.5]undecane, Spiroacetal 5: (*E*,*E*)-2-propyl-8-methyl-1,7-dioxaspiro[5.5]undecane.

GC–MS analysis of rectal glands showed that solvent was a significant factor for the amounts of compounds (solvent: F_2, 594_ = 66.653, *P* < 0.001, compound: F_21, 594_ = 118.087, *P* < 0.001, solvent × compound: F_41, 594_ = 4.576, *P* < 0.001). DCM and *n*-hexane generally extracted similar amounts of esters, and both generally extracted greater amounts than ethanol (Fig. 1A, Table S2). The overall patterns of solvents are similar for the compounds although the lower efficiency of ethanol was less evident for compounds that were less abundant overall in all solvents, notably methyl laurate, methyl myristate, methyl palmitoleate and diethyl succinate (Table S2, Figure S1). Ethanol extracts typically did not contain ethyl isobutyrate and ethyl 2-methylbutanoate, which were detected in the DCM and *n*-hexane extracts.

The DCM extracts consistently contained greater amounts of amides than either *n*-hexane or ethanol (Fig. 1B, Table S2). There was no difference between *n*-hexane and ethanol in amounts of amides (Table S2). In case of spiroacetals, there was no significant difference between *n*-hexane, DCM and ethanol except for the most abundant spiroacetal, (*E*,*E*)-2,8-dimethyl-1,7-dioxaspiro[5.5]undecane for which *n*-hexane extracted greater amounts than DCM (*P* < 0.001) or ethanol (*P* < 0.001), while DCM extracted greater amount than ethanol (*P* = 0.001).

#### Effect of crushing glands

**Figure 2:**
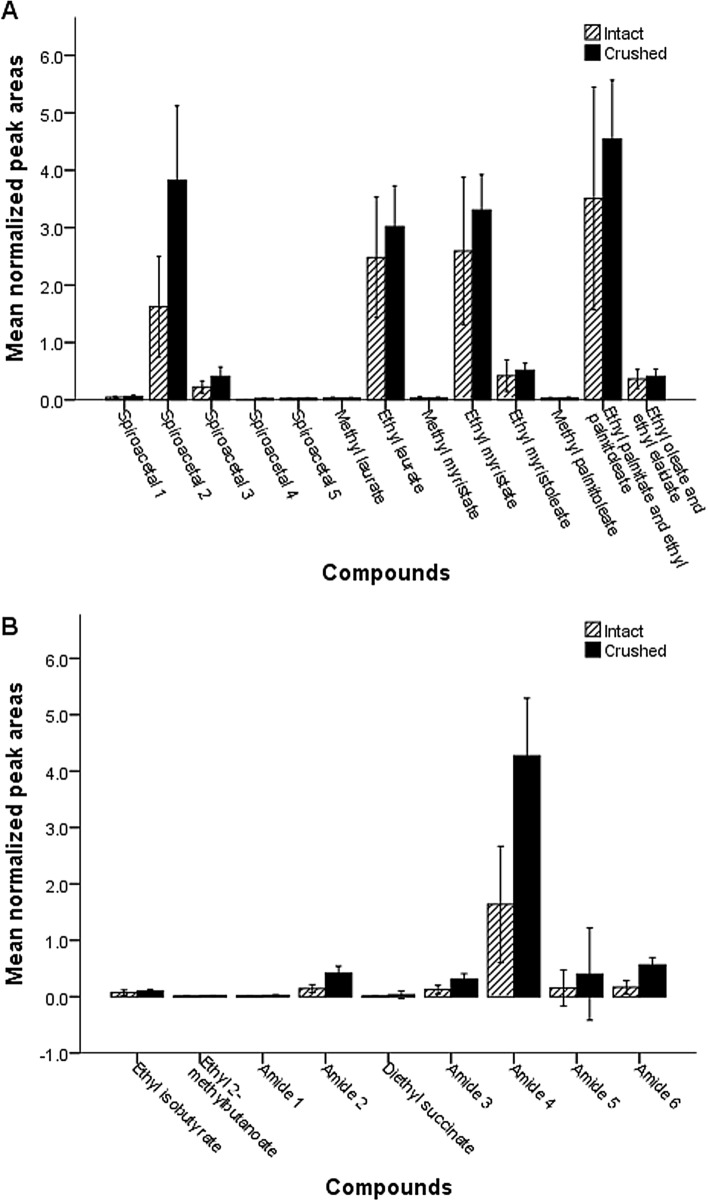
Graphical display of mean normalized peak areas (*n* = 10) obtained for rectal glands extract of female (**A**) and male (**B**) *Bactrocera tryoni* using crushed rectal glands and non-crushed rectal glands. Error bars represent the confidence interval for the mean at 95% confidence level. Amide 1: *N*-(2-methylbutyl)acetamide, Amide 2: *N*-(3-methylbutyl)acetamide, Amide 3: *N*-(2-methylbutyl)propanamide, Amide 4: *N*-(3-methylbutyl)propanamide, Amide 5: *N*-(2-methylbutyl)isobutyrate, Amide 6: *N*-(3-methylbutyl)isobutyrate, Spiroacetal 1: 2,7-dimethyl-1,6-dioxaspiro[4.5]decane, Spiroacetal 2: (*E*,*E*)-2,8-dimethyl-1,7-dioxaspiro[5.5]undecane, Spiroacetal 3: (*E*,*E*)-2-ethyl-8-methyl-1,7-dioxaspiro[5.5]undecane, Spiroacetal 4: (*E*,*E*)-2-ethyl-2,8-dimethyl-1,7-dioxaspiro[5.5]undecane, Spiroacetal 5: (*E*,*E*)-2-propyl-8-methyl-1,7-dioxaspiro[5.5]undecane.

Crushing of rectal glands significantly increased extraction of compounds (crushing: F_1, 396_ = 65.862, *P* < 0.001, compound: F_21, 396_ = 159.600, *P* < 0.001, crushing × compound: F_21, 396_ = 6.171, *P* < 0.001). The overall patterns of crushing are similar for the compounds (Figure S2) although the higher efficiency of crushing glands was less evident for compounds that were less abundant overall in male and female rectal glands, notably ethyl isobutyrate, ethyl 2-methylbutanoate, diethyl succinate, *N*-(2-methylbutyl)acetamide, *N*-(2-methylbutyl)propanamide, *N*-(2-methylbutyl)isobutyrate, methyl laurate, methyl myristate, methyl palmitoleate, ethyl myristoleate and ethyl oleate and ethyl elaidate (Fig. 2, Table S3).

## Effect of SPME fibers in static headspace sampling

**Figure 3:**
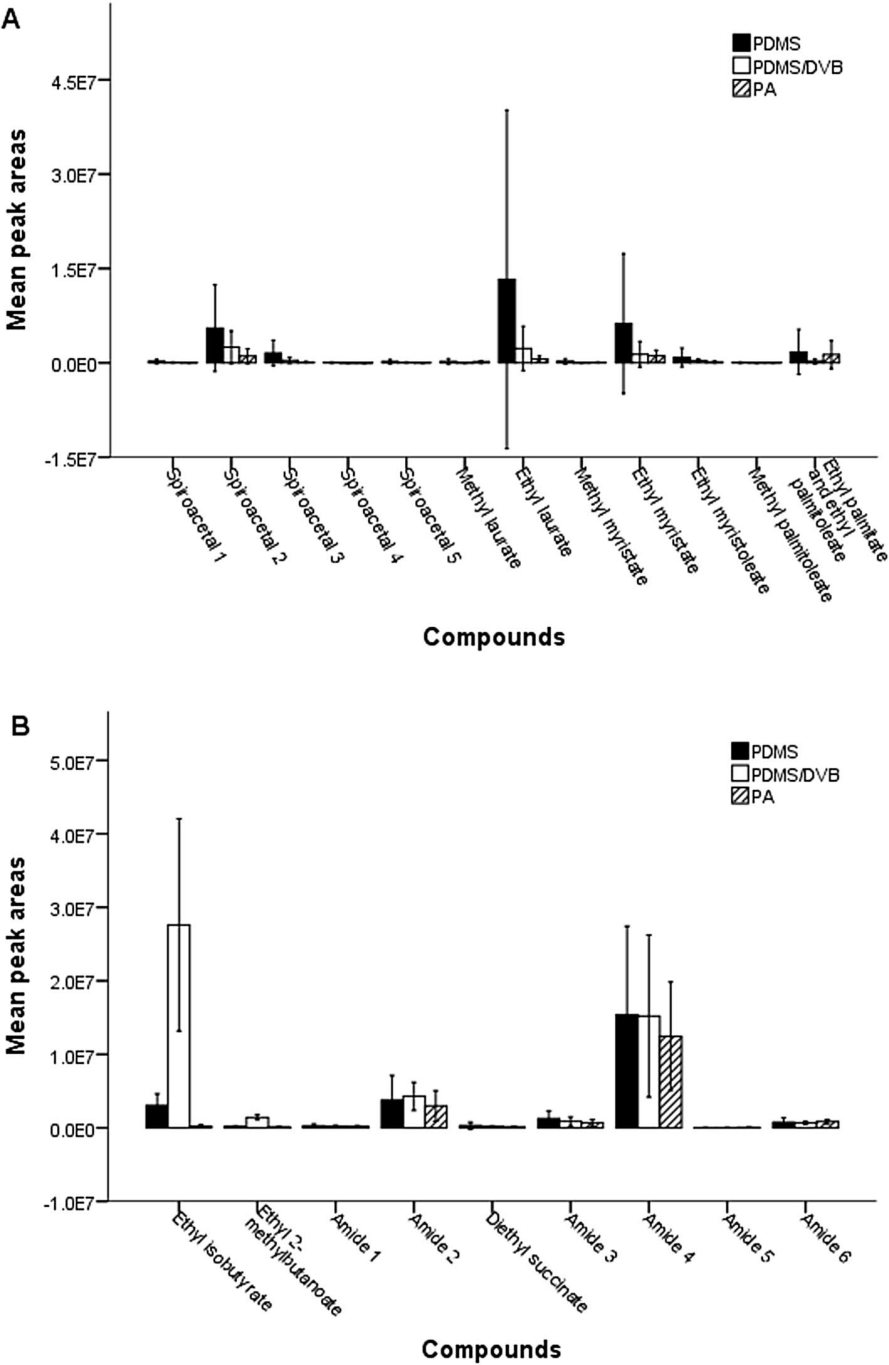
Graphical display of mean peak areas (*n* = 6) obtained for headspace collection of female (A) and male (B) *Bactrocera tryoni* using three SPME fibers, polydimethylsiloxane (PDMS), poly(dimethylsiloxane)–divinylbenzene (PDMS–DVB) and polyacrylate (PA). Error bars represent the confidence interval for the mean at 95% confidence level. Amide 1: *N*-(2-methylbutyl)acetamide, Amide 2: *N*-(3-methylbutyl)acetamide, Amide 3: *N*-(2-methylbutyl)propanamide, Amide 4: *N*-(3-methylbutyl)propanamide, Amide 5: *N*-(2-methylbutyl)isobutyrate, Amide 6: *N*-(3-methylbutyl)isobutyrate, Spiroacetal 1: 2,7-dimethyl-1,6-dioxaspiro[4.5]decane, Spiroacetal 2: (*E*,*E*)-2,8-dimethyl-1,7-dioxaspiro[5.5]undecane, Spiroacetal 3: (*E*,*E*)-2-ethyl-8-methyl-1,7-dioxaspiro[5.5]undecane, Spiroacetal 4: (*E*,*E*)-2-ethyl-2,8-dimethyl-1,7-dioxaspiro[5.5]undecane, Spiroacetal 5: (*E*,*E*)-2-propyl-8-methyl-1,7-dioxaspiro[5.5]undecane.

The type of SPME fiber affected the amounts of compounds trapped from female and male headspace samples (fiber: F_2, 315_ = 7.636, *P* = 0.001, compound: F_20, 315_ = 11.041, *P* < 0.001, fiber × compound: F_40, 315_ = 2.622, *P* < 0.001) (Fig. 3 A and B). PDMS trapped more spiroacetals than PA (Figure S3, Table S4). There was no significant difference between PDMS/DVB and PA or PDMS (Table S4). The type of fiber did not affect the amounts of amides and esters except for the two lighter esters, ethyl isobutyrate and ethyl-2-methylbutanoate. PDMS/DVB trapped more ethyl isobutyrate and ethyl-2-methylbutanoate than PDMS (*P* = 0.002 and *P* = 0.005, respectively), and both PDMS/DVB and PDMS trapped more than PA (Table S4).

### Effect of sorbent material and time in dynamic headspace sampling

Analysis of headspace samples revealed that the type of sorbent material and sampling duration significantly affected the amounts of compounds trapped (sorbent: F_1, 950_ = 11.727, *P* = 0.001, time: F_4, 950_ = 170.902, *P* < 0.001, compound: F_18, 950_ = 945.192, *P* < 0.001, sorbent × time: F_4, 950_ = 8.605, *P* < 0.001, sorbent × compound: F_18, 950_ = 64.468, *P* < 0.001, time × compound: F_72, 950_ = 20.828, *P* < 0.001, sorbent × time × compound: F_72, 950_ = 4.144, *P* < 0.001).

**Figure 4:**
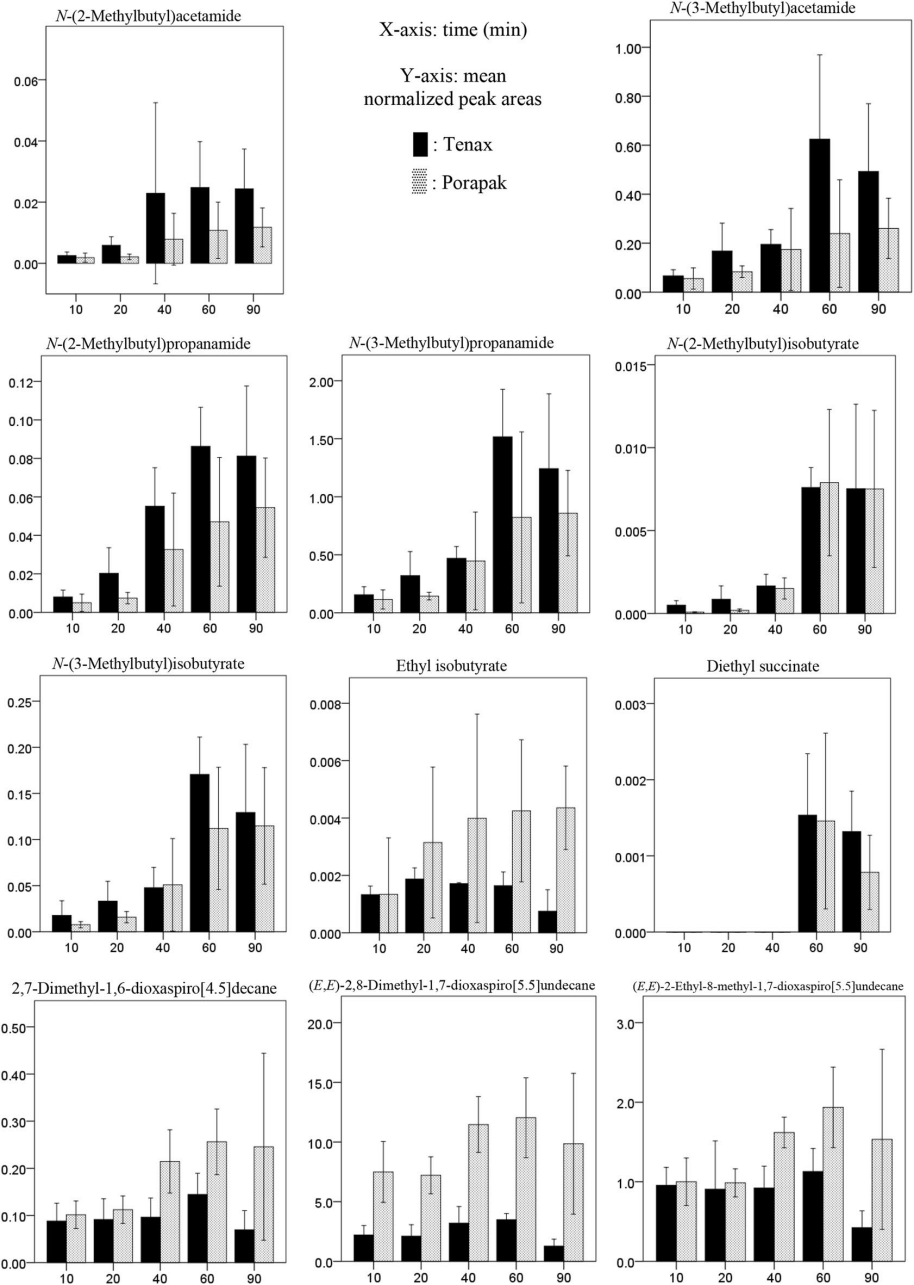

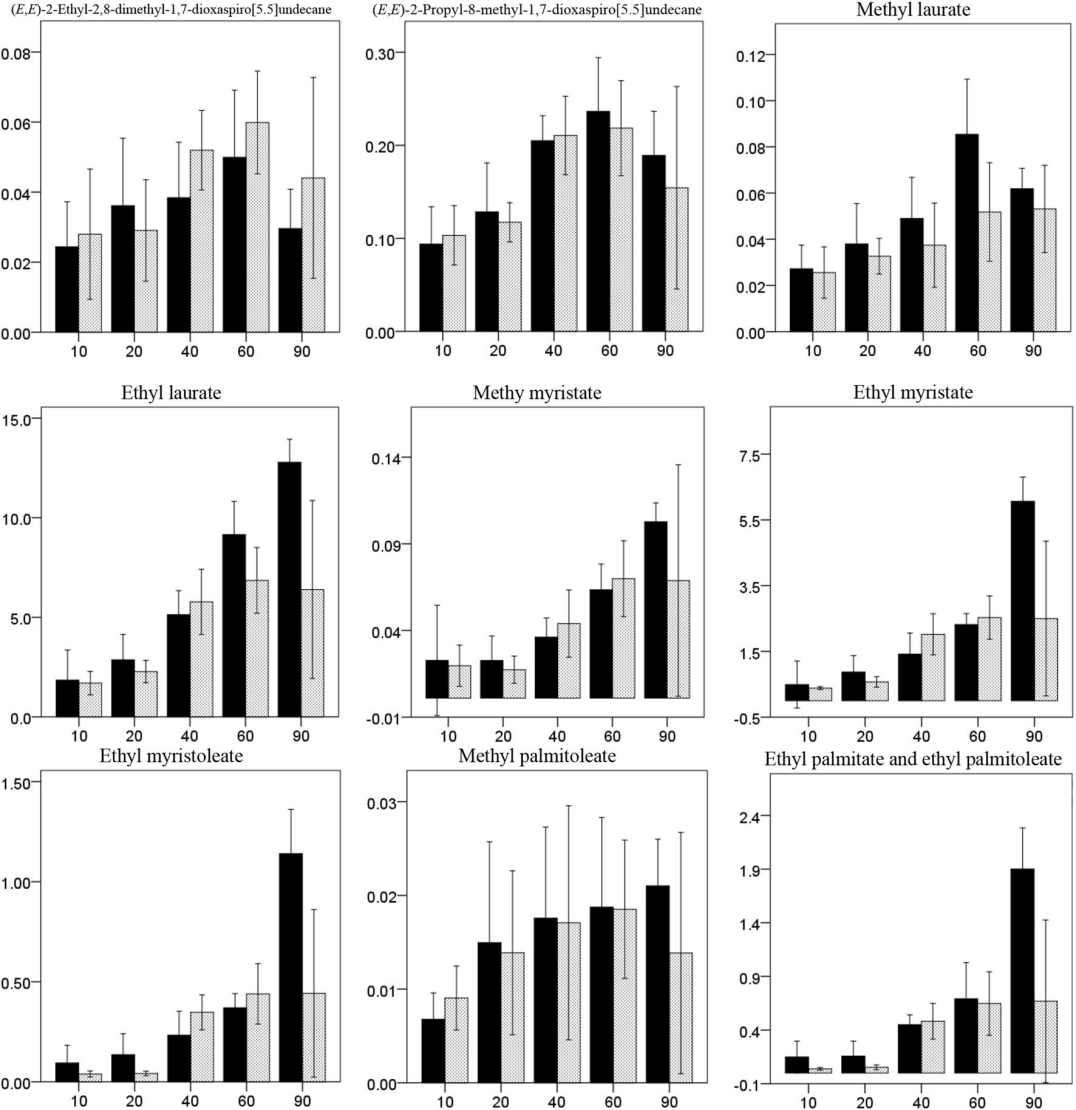
Graphical display of estimated mean normalized peak area (*n* = 6) obtained for headspace collection of male and female *Bactrocera tryoni* using two polymer sorbents, Tenax-GR and Porapak Q. Compounds obtained from males include: *N*-(2-methylbutyl)acetamide, *N*-(3-methylbutyl)acetamide, *N*-(2-methylbutyl)propanamide, *N*-(3-methylbutyl)propanamide, *N*-(2-methylbutyl)isobutyrate and *N*-(3-methylbutyl)isobutyrate, ethyl isobutyrate and diethyl succinate. Compounds obtained from females include: 2,7-dimethyl-1,6-dioxaspiro[4.5]decane, (*E*,*E*)-2,8-dimethyl-1,7-dioxaspiro[5.5]undecane , (*E*,*E*)-2-ethyl-8-methyl-1,7-dioxaspiro[5.5]undecane, (*E*,*E*)-2-ethyl-2,8-dimethyl-1,7-dioxaspiro[5.5]undecane, (*E*,*E*)-2-propyl-8-methyl-1,7-dioxaspiro[5.5]undecane, methyl laurate, ethyl laurate, methyl myristate, ethyl myristate, ethyl myristoleate, methyl palmitoleate, ethyl palmitate and ethyl palmitoleate. Error bars represent the confidence interval for the mean at 95% confidence level.

For spiroacetals, while Porapak trapped more 2,7-dimethyl-1,6-dioxaspiro[4.5]decane, (*E*,*E*)-2,8-dimethyl-1,7-dioxaspiro[5.5]undecane and (*E*,*E*)-2-ethyl-8-methyl-1,7-dioxaspiro[5.5]undecane (spiroacetals 1, 2 and 3) than Tenax, this was not the case for (*E*,*E*)-2-ethyl-2,8-dimethyl-1,7-dioxaspiro[5.5]undecane and (*E*,*E*)-2-propyl-8-methyl-1,7-dioxaspiro[5.5]undecane (spiroacetals 4 and 5) (Fig. 4, Table S5 and S6). Generally, the amounts of spiroacetals increased with sampling time for both Tenax and Porapak until 60 min (Fig. 4). However, there were some subtle differences among compounds in the relationship between time and amount (Table S5).

While Porapak and Tenax trapped very similar amounts of most esters, Tenax trapped significantly more ethyl laurate, ethyl myristate, ethyl myristoleate and ethylpalmitate/palmitoleate than Porapak at 90 min (Fig. 4, Table S6). Generally, the amounts of long chain esters increased with sampling time for Tenax, while they increased until 60 min for Porapak (Fig. 4). However, there were some subtle differences among compounds in the relationship between time and amount (Table S5 and S6).

Tenax generally collected higher amounts of amides than Porapak (Fig. 4). The amounts of amides increased with sampling time until 60 min (Fig. 4). The overall patterns are similar for the different amides, with only subtle differences (Fig. 4, Table S6). Both Tenax and Porapak trapped trace amounts of diethyl succinate and ethyl 2-methylbutanoate, but the amounts of these compounds were not able to be statistically analyzed.

## Discussion

### Rectal gland volatiles of Queensland fruit fly

Adding to the six aliphatic amides that have been reported previously in the rectal glands of male *B. tryoni*^37^, we here describe an additional six previously unreported compounds in rectal glands and emissions of male *B. tryoni*. While ethyl isobutyrate has been tentatively identified in the volatiles of male *B. tryoni* by Kumaran et al.^43^ it was not reported as a rectal gland component. Kumaran et al.^43^ also tentatively identified only 3 amides as components of rectal glands including, *N*-(3-methylbutyl)acetamide, *N*-hexylpropanamide and *N*-propylbutyramide, two of which are different from those reported by Bellas and Fletcher^37^ and the present study. Of these, *N*-(3-methylbutyl)acetamide) and *N*-hexylpropanamide, as well as 2-hydroxypropanamide and two propanoic acid derivatives, 2-methyl propanoic acid and 2-methylundecyl propenoate, were also reported in the volatiles released by male *B. tryoni* by Kumaran et al.^43^ Although pheromone composition may be affected qualitatively and quantitatively by larval diets^49^, the additional compounds found in the present study have most likely been overlooked previously. The ethyl and propyl esters are highly volatile and may have been lost through volatilization during extraction or trapping^50^. The four short chain esters—ethyl propanoate, ethyl 2-methylbutanoate, propyl isobutyrate and ethyl 2-methylpentanoate – are here identified for the first time in fruit flies. Some of these compounds have been reported previously in fruits. For example, ethyl propanoate, which has a pineapple-like odor^51^, is a common volatile in many ripe fruits that attract *B. tryoni* females including mango and apple^52^. Ethyl propanoate has also been considered as an attractant for other frugivorous pest insects. For instance, ethyl propanoate increases attraction of the African palm weevil *Rhynchophorus phoenicis* to aggregation pheromone^53^. Ethyl isobutyrate has been reported as an important contributor to the sweet aroma of fresh pineapple^54^ and other fruits including apple^55^, orange^56^ and berries^57,58^. Ethyl isobutyrate has been also reported as a strong electrophysiologically active compound for the female blueberry fruit fly, *Rhagoletis mendax*^57^. Ethyl 2-methylbutanoate is found in apples^55^, pineapples^59,60^, oranges^61^, and berries^57,58^. Diethyl succinate has been found in rectal glands of male *B. halfordiae*^15^ and *B. kraussi*^62^. This compound is known as an attractant for the spotted wing Drosophila, *Drosophila suzukii*^63^. The strong sweet smell of volatiles released by *B. tryoni* males during sexual activity does not resemble that of the amides described previously by Bellas and Fletcher^37^ but does resemble that of the esters reported in the present study.

In female *B. tryoni*, we found three compounds that have not been reported previously^19,38^. While ethyl (9,12)-octadecadienoate was not detected in our rectal gland extracts, despite being reported in a previous study^38 ^, *N*-(2-methylbutyl)acetamide, propyl laurate and methyl palmitoleate were detected for the first time. These are all present at low concentrations and this likely explains why they were not detected previously. It is possible that differences between studies in the reporting of these compounds is a result of differences in rearing conditions and especially larval diet^49^.

### Rectal glands

Although relying only on rectal gland extraction can mean that volatiles released from elsewhere on the flies are missed, this is a practical, rapid and selective way to collect compounds that fruit flies emit from these glands^7,8^ and is important for confirming the source of compounds collected in headspace samples. We assessed the effectiveness of three different solvents for rectal gland extractions, including the non-polar *n*-hexane, medium-polarity DCM and polar ethanol. In general, under the conditions used in this study, GC–MS of ethanol extracts showed very incomplete mass profiles for ethyl isobutyrate and ethyl 2-methylbutanoate. It was difficult to identify these compounds without the assistance of the *n*-hexane and DCM extracts. Since ethanol is the most polar solvent employed, it is possible that ethanol did not absorb the volatile esters during the extraction time used in this study. DCM and *n*-hexane extracted similar concentrations of lower volatility long chain fatty acid esters but there were differences in the extraction of more volatile compounds. DCM was the most effective solvent for extraction of amides, whereas there was no significant difference between the three solvents for the extraction of spiroacetals except for (*E*,*E*)-2,8-dimethyl-1,7-dioxaspiro[5.5]undecane that *n*-hexane was more effective. Extracts from crushed rectal glands contained the same compounds as those from intact glands, but at larger amounts. This shows that there is a benefit to crushing glands, and importantly also demonstrates that studies where extracts were taken from intact rectal glands are at least qualitatively comparable to those extracts obtained from crushed glands as no compounds were missed in intact gland samples.

### Headspace

The selection of fiber is a critical aspect of using SPME. The three SMPE fibers used in this study exhibited different performances. In general, the most polar fiber, PA, was found to be inefficient at collecting spiroacetals, ethyl isobutyrate and ethyl-2-methylbutanoate. PDMS was found to have better or at least the same performance as PMDS/DVB for collection of the more polar and volatile compounds except for ethyl isobutyrate and ethyl-2-methylbutanoate . The different concentration of analyte on the fiber may result from several factors including the chemical properties of the analyte, the equilibrium time^64,65^, the experimental conditions (temperature and humidity)^66^, and storage conditions^67^.Of these factors, only the properties of the analytes would have affected the outcomes in this study because the experiments were conducted in controlled environment rooms (25 ± 0.5 °C, 65 ± 5% RH), and used the same equilibrium time and storage conditions. The short chain esters are volatile but have polar surfaces that would require sorbent affinity with volatile and slightly polar properties. PDMS/DVB would be suitable for such applications, and this is consistent with our results. Because of the substantial differences amongst headspace compounds in collection efficiency on different SPME fibers, there is a particular need for care in both the selection of fibers and in the interpretation of analyzed samples.

In dynamic headspace sampling, Porapak was found to be more effective for spiroacetals, while Tenax was more effective for esters and amides. Based on sorbent properties these results were anticipated. Sampling period and flow rate are also important factors. Tenax started to lose the six amides, diethyl succinate and all spiroacetals once air was passed through for 60 min (Fig. 4). Porapak showed similar capacity for *N-*(2-methylbutyl)isobutyramide, all spiroacetals and esters (except methyl laurate, ethyl myristoleate and ethyl palmitate/palmitoleate). Porapak showed greater capacity to retain the other five amides, methyl laurate, ethyl myristoleate and ethyl palmitate/palmitoleate after 60 min, whereas Tenax showed greater capacity to retain all the long chain esters after 60 min (Fig. 4).

Active and passive headspace sampling techniques each have advantages and disadvantages. Advantages of SPME include higher sensitivity, ease of handling, shorter adsorption time, no solvent peak in GC and easy sequential sampling. However, quantitation of analytes in many types of SPME matrices is a major challenge^67–69^. The other disadvantage is that the sample can be used only once^67^. While dynamic headspace sampling can have lower sensitivity, quantitation of analytes can be conveniently achieved as the calibration of a compound using an internal standard is easily achieved in liquid samples. Liquid samples can also be re-used, in their original form or after concentration or dilution if needed, and can also be used later for other assays (*e.g.* electrophysiological assays or bioassays)^70^. A disadvantage is that if samples contain highly volatile compounds, the solvent peak may mask these compounds in GC. Overall, dynamic headspace methods are more effective for quantitation, while SPME has some advantages for qualitative analysis.

In brief, each sampling technique may bias the interpretation of rectal gland contents or released volatile compositions. Therefore, it is important to consider differences that can be introduced by sampling techniques when interpreting volatiles of tephritid fruit flies, and especially when comparing studies that have used different techniques. There are advantages and disadvantages for each method, and it may often be useful to employ more than one method to ensure comprehensive sample collection and analysis.

## Supplementary information

Supplementary information.
